# Experiences of COPD patients with existing smoking cessation programs and their preferences for improvement - a qualitative analysis

**DOI:** 10.1186/s12971-016-0097-4

**Published:** 2016-08-24

**Authors:** I. Aumann, L. Tedja, J. M. Graf von der Schulenburg

**Affiliations:** 1 Center for Health Economics Research Hannover (CHERH), Leibniz University of Hannover, Otto-Brenner-Str. 1, 30159 Hannover, Germany; 2 Biomedical Research in Endstage and Obstructive Lung Disease Hannover (BREATH), Member of the German Center for Lung Research (DZL), Hannover, Germany

**Keywords:** Chronic obstructive pulmonary disease, COPD, Smoking cessation, Preferences, Qualitative interviews

## Abstract

**Background:**

Smoking is a major risk factor for chronic obstructive pulmonary disease (COPD). For current smokers who are diagnosed with COPD, their first treatment option is to stop smoking. Motivation is necessary for long-term smoking cessation; therefore, when designing smoking cessation programs, the patients’ needs and preferences should be considered. We focused on COPD patients’ experiences with existing smoking cessation programs and evaluated their preferences for the improvement of these programs.

**Methods:**

We conducted 18 guideline-based interviews with COPD patients between April and June 2014 in Germany. Each patient with COPD, who was a current or past smoker and had made at least one attempt to quit smoking in the past 5 years, was included in the study. We audiotaped, verbatim transcribed, and evaluated the interviews, using content analysis.

**Results:**

The patients had broad and different experiences with pharmaceutical, behavioral, and alternative approaches that supported or negatively influenced the smoking cessation process. Pharmaceuticals were viewed as an expensive alternative with many side effects although they helped to stop cravings for a few moments. Furthermore, the bad structure and impersonal content of the seminars for smoking cessation negatively influenced group cohesion, and therefore degrading the patients’ motivation to stop smoking. Alternative methods, such as acupuncture and hypnosis were mostly ineffective in smoking cessation, but in some cases, served as motivational strategies.

**Conclusion:**

Negative experiences with smoking cessation were explained by the patients’ lack of motivation or resolution. Other negative experiences, such as the structure of seminars for smoking cessation and the high price of pharmaceuticals should be addressed through policy changes to increase the patients’ motivation to quit smoking.

## Background

The Global Burden of Disease Study estimates that chronic obstructive pulmonary disease (COPD) will become the third leading cause of death worldwide by 2020 [1]. The Global Initiative for Chronic Obstructive Lung Disease defines COPD as ‘a common preventable and treatable disease which is characterized by persistent airflow limitation that is usually progressive and associated with an enhanced chronic inflammatory response in the airways and the lung to noxious particles or gases’ [2]. An important risk factor, although not the only one, for the high prevalence of COPD in Germany and other industrial nations is smoking. According to the estimates by the World Health Organization, 73 % of COPD-related cases of death are caused by smoking [3]. The percentage of lifelong smokers who develop COPD is 40–50 % [4].

Another important consequence of COPD is the economic burden. According to estimations in the European White Book, the amount spent on COPD is approximately 38.6 billion Euros, which represents 56 % of the total direct costs of respiratory diseases [4]. COPD is not curable; however, stopping smoking is the most effective way to prevent the exacerbation of the disease and to maintain a state of health. Hence, after a COPD diagnosis, smoking cessation is the first and most important treatment option [2].

Nevertheless, smoking cessation can be very difficult for patients with COPD, and there are no single factors that predict long-term success. Different behavioural and pharmaceutical interventions support smoking cessation [5]. Behavioural interventions encompass psychological counselling (face-to-face or telephone), self-help measures (books, apps, and the Internet), individual or group therapy, and alternative remedies, such as acupuncture. Nicotine replacement therapy (NRT), bupropion, and varenicline are examples of pharmaceutical interventions. Many support programs may increase COPD patients’ motivation to stop smoking and their chances for success. However, it is unclear if these programs correspond to the preferences of COPD patients who wish to stop smoking and if these interventions changed the patients behavior. Michi et al. identified different sources of behaviour that could prove fruitful targets for intervention [6]. According to this information Michi et al. developed the COM-B framework which is based on an interacting system involving the components of capability (C), opportunity (O), motivation (M) and behavior (B). Therefore this framework shows that the success of interventions for smoking cessation is based on different perspectives. In the literature different qualitative studies have evaluated the factors that do and do not motivate smokers with COPD to stop smoking [7–10]. Wilson [7] and van Eklund [9] et al. reported reasons why patients with COPD did not quit smoking, which included difficulty breaking lifelong habits and feeling that it was too late for them to stop smoking. The patients also reported that the demands of other people could lead to the continuation of smoking. Support from relatives and care providers during smoking cessation were welcomed by these patients but they wanted to make the decision to quit on their own. Two studies conducted by van Eerd et al. analysed the differences in smoking and quitting behaviours between patients with and those without COPD [8, 10]. One of these studies concluded that the smokers with COPD did not believe in the efficacy of smoking cessation aids, although these smokers used the different aids more often than the smokers without COPD did [8, 10]. Nevertheless no study compared patients with COPD who had successfully stop smoking with patients who are unable to quit smoking. Therefore it is important to compare the preferences and motivating aspects for different smoking cessation intervention in both groups to identify factors that explain success and failure of interventions. Smoking cessation interventions also differ in structure and reimbursement systems between industrial nations. For Germany there is lack of studies that give a broad overview about the experiences and preferences of patients with COPD for different forms of smoking cessation interventions.

The aim of the present study was to analyse the experiences of smokers and former smokers with COPD with existing smoking cessation programs and to evaluate the smokers’ preferences for the improvement of the programs. These preferences will be classified into the three components of the COM-B Model to analyse the different factors that could influence the behaviour of patients with COPD. Therefore, we chose to interview patients with COPD who were current or former smokers and who had tried to quit smoking in the past 5 years.

## Methods

### Study design and population

To elicit patients’ personal experiences and their views on the smoking cessation process, we used a qualitative research design, as it focuses on the subjective perspective of the observed.

This study was approved by the Committee for Clinical Ethics of the Hannover Medical School (MHH), Germany (2214-2014). We conducted semi-structured, guideline-based interviews with COPD patients, and we recruited the study’s sample by asking primary care physicians, pulmonologists, and university clinics to distribute information flyers and post requests for participation on COPD social-networking sites. Patients who were current smokers or had smoked previously and who had made at least one attempt to stop smoking in the past 5 years were recruited. We decided to use the time frame of the past 5 years because a longer time frame would have made it difficult for the patients to recall their experiences in sufficient detail. To capture different experiences from all the patients in this study, we maximized the variation in our sample by including men and women from different social backgrounds and ages, who had used different smoking cessation methods (e.g. medication, group counselling, hypnosis). Because of the time frame of the project and the patients’ wishes, a research assistant from the institute (Tedja) conducted four interviews in the patients’ homes and 14 interviews via telephone. The number of interviews was not predefined. We always conducted three to four interviews and coded them first before we interviewed the next four patients. Therefore, we stopped conducting interviews after no new information emerged. Prior to conducting the interviews, we obtained written consent from the patients and gave them additional information about the project, via mail. The confidential and anonymous handling of all personal data was assured. We provided information to the participants about the study’s aim, the voluntary nature of their consent, and the procedures for data collection and processing. The research assistant conducted all the interviews between April and June 2014.

### Guidelines

Based on information from the literature and input from an interdisciplinary group of researchers, we structured the interviews with the use of two guidelines. These guidelines were slightly different for smokers and ex-smokers and were developed according to the COM-B framework for understanding behavior [6]. The interviews consisted of open-ended questions that encouraged the COPD patients to talk about their experiences and preferences regarding the different smoking cessation processes in their own words. The guideline-based questions focused on the patients’ experiences with smoking cessation therapies that they had undergone. The interview started with questions regarding their motivation to stop smoking. Therefore, the patients were asked to describe a situation in which they wanted to quit smoking. Further questions pertained to their chosen smoking cessation method, and the reasons for their selection were scrutinized. During this phase, possible reasons for choosing different forms of smoking cessation, such as the effect of other people, the costs, and time-related reasons were explored. We also asked the patients about specific aspects of the smoking cessation methods that helped them make the decision to quit, what was comfortable, and what kind of support they needed in order to deal better with the smoking cessation situation. In the last session of the interview, smokers described the characteristics that a smoking cessation program should not have.

Each question in the guidelines provides information about the factors (Capability, Opportunity, and Motivation) that influence and describe the respondents’ behavior [6]. These factors are part of the COM-B framework for understanding behavior, which was developed by Michie et al. The framework hypothesizes that the interaction among the three components influences the performance of a behavior. Each component may influence behavior directly. Thus, Opportunity and Capability might influence Motivation, which also affects behavior.

Each factor influences one another in a different manner. Information about capability (psychological and physical capacity) becomes apparent during the descriptions of a typical situation related to smoking cessation. The necessary knowledge and skills of the patients about the different ways to stop smoking also may be identified during the interviews. Therefore, we first asked the patients to talk about a situation related to their last attempt to quit smoking. Only towards the end of describing the situation did we ask the patients about possible experiences with different methods that were not mentioned before. The questions also focused on the patients’ motivation or the brain processes that energized a direct behavior. This information was obtained by asking questions about the reasons for choosing certain smoking cessation therapies and from the patients’ descriptions of their experiences with these methods. The last factor, Opportunity, describes all external factors that make the behavior possible for the patients [6]. During the interviews, we asked the patients, if they had support during the smoking cessation process. We used the COM-B framework to understand why some patients with COPD used special smoking cessation aids and others did not. We also wanted to analyze the influence of the experiences on each of the three factors.

### Methods of qualitative analysis

The interviews lasted approximately 1 h, and we audiotaped and verbally transcribed them. One research assistant who was not involved in the interviews, transcribed them, and another assistant reviewed the audio recordings and transcripts to verify the accuracy of the first transcription. As the interviews were conducted in German, the citations were translated by two professional translators who are native speakers. Disparities were clarified bilaterally. Furthermore, each interview was completely anonymous.

We analysed the data using content analysis methods. The analysis involved interpreting and paraphrasing relevant text independently through content analysis with the inclusion of inductive categories [11, 12]. To ensure accuracy of the analysis, two researchers (Tedja and Aumann) independently read the interviews, paraphrased the relevant text using the MAXQDA program, and generated a preliminary codebook. First, the researchers analysed the text based on deductive categories, which we derived from the questions in the guideline. The inductive categories were developed independently by each interviewer from the content of the interview. Disagreements were discussed between the two researchers and resolved by consensus. In order to obtain an overall impression of the content of the interviews, the researchers read and re-read the transcripts and revised the coding system accordingly. In subsequent discussions, the researcher checked the codes for consistency and agreement, and resolved any differences by an iterative process. The aim of a content analysis is to identify cross-relationships, repetitions, commonalities, and differences in the statements to detect any trends regarding the results. To achieve this, all interpretations and arguments were documented and supported by citations.

## Results

Eighteen COPD patients (seven men; 11 women) participated in the interviews. We stopped the interviews when the main elements were repeated in the last three interviews. The average age of the patients at the time of the interviews was 57 years. Five of the 18 patients still smoked and the average age at which the patients began smoking was 17 years. Table 1 presents the patients’ characteristics in detail.

**Table 1:** Evaluation of the socioeconomic questionnaire

No.	Sex	Age	Marital status	Age of starting smoking	Years since quit	Number of years smoked	Profession
1	Female	53	Married	35	3	15	Office clerk
2	Male	62	Married	21	2	39	Banker with management position
3	Female	54	Widowed	14	Current smoker	14	Bookseller
4	Male	50	Married	15	4	31	Branch manager
5	Female	59	Single	17	3	39	Nurse
6	Female	65	Widowed	20	Current smoker	45	Insurance clerk
7	Male	58	Married	13	2	43	Surveying technicians
8	Female	56	Married	15	1	40	Geriatric nurse
9	Female	53	Married	16	1	36	Notary clerk
10	male	54	Married	19	1	34	Professional soldier
11	Female	49	Married	14	1	34	Tax officer
12	Female	59	Married	14	4	41	Nurse
13	male	59	Relationship	15	Current smoker	44	Office machine repairer
14	Female	56	Divorced	17	5	34	Office clerk
15	male	59	Married	14	2	43	Seller
16	female	59	Widowed	15	Current smoker	44	Tax officer
17	male	59	Single	14	1	44	Project leader
18	female	64	Relationship	17	Current smoker	47	Postal clerk

With regards to the research questions, we obtained information about the patients’ experiences with pharmaceutical, behavioral, and alternative smoking cessation therapies, and their preferences for improving the smoking cessation process.

### Experiences with pharmaceutical support

Three different pharmaceutical therapies for smoking cessation - varenicline, NRT, and bupropion - are available for patients in Germany. Most of the interviewed patients had heard about the different forms of NRT, especially the gum and patch (see Table 2). The medication bupropion was less familiar than varenicline. Some patients had tried varenicline therapy and nearly all had used some form of NRT; however, none of them had tried bupropion. All the patients who had tried varenicline agreed that its high cost and powerful side effects, such as nausea and the more rarely reported side effects of deafness and loss of body control, were significant disadvantages.

**Table 2:** Information about medications

No.	Have heard about …					Have used …		
	NRT (Nikotin)			Bupropion	Vareniclin	NRT	Bupropion zyban	Vareniclin champix
	Patches	Spray	Gum					
1	✓	X	✓	X	✓	✓ (patches, gums)	X	X
2	✓	✓	✓	X	X	✓ (patches, gums)	X	X
3	✓	X	✓	✓	✓	✓ (patches, gums)	X	X
4	✓	✓	✓	X	X	✓ (patches, gums)	X	✓
5	X	X	✓	X	X	✓ (gums)	X	X
6	✓	✓	✓	X	X	✓ (patches)	X	X
7	✓	X	✓	X	X	✓ (patches)	X	X
8	✓	X	✓	X	✓	✓ (patches, spray)	X	✓
9	✓	✓	X	X	X	X	X	X
10	✓	X	✓	✓	✓	X	X	X
11	✓	X	X	X	X	X	X	X
12	X	X	X	✓	✓	X	X	✓
13	X	X	X	X	X	X	X	X
14	✓	n.a.	✓	X	X	✓ (gums)	X	X
15	✓	✓	✓	X	X	✓ (gums)	X	X
16	✓	n.a.	✓	X	n.a.	X	X	X
17	✓	✓	✓	X	✓	✓ (gums)	X	X
18	✓	✓	X	X	✓	✓ (patches)	X	✓

✓ have heard about the medication or have used it

X not known before or not used before

*n.a.* not available

*“I found it [Vareniclin] absolutely terrible. I had the feeling that I was under the influence of drugs, you know?” (No. 8, f, 56 years)*

*“There was just the disadvantage that I was spending money on something that didn’t help me.” (No. 4, m, 50 years)*

Patients provided varied feedback regarding the efficacy of treatment with varenicline. Some patients had successfully stopped smoking, and one patient reported a reduction in smoking cravings.

*“And then I noticed: It works! I don’t smoke anymore. And then, from one moment to the next … I stopped smoking.” (No. 12, f, 59">years*)

Apart from therapy with varenicline, patients also had various experiences with NRT in the form of nicotine patches and nicotine chewing gum. None of the patients had tried other forms of NRT, such as lozenges, inhalers, or nasal sprays. For one patient, who used the nicotine patch, the dosage was so low that the person unintentionally continued smoking while they were wearing the patch. Another patient who had tried the nicotine patch was smoke free for 30 months. The main limitations of the nicotine patch that was cited by the patients were their high cost, the side effect of a tingling sensation in the hands, and the daily changes in the skin that came into contact with the patch. According to the patients, although the nicotine chewing gum was ineffective in helping them stop smoking completely, it did help them take a deep breath, and thereby attenuate smoking cravings.

*“These (nicotine) chewing gums … and at that moment, you get about half an hour’s breathing room: three quarters of an hour in which you really don’t gasp for a cigarette, but rather can really let out a deep breath and say, “Oh thank God, peace at last.” (No. 7, m, 58 years*)

In particular, the users of nicotine chewing gum frequently cited a bad taste and stronger cigarette cravings as limitations of this form of NRT. In addition, some patients mentioned experiencing an accelerated pulse and tingling sensation in the hands. However, they stated that the therapy was easy to use.

*“Yes, but the disadvantages are that they taste so disgusting hey? That one has to chew them for about five minutes and can then throw them away – they have such an awful aftertaste.” (No. 14, f, 56 years)*

The interviews revealed differences between the current smokers and ex-smokers. Both groups often used nicotine substitutes during the smoking cessation process. It was found that the ex-smokers often combined the use of nicotine substitutes with a strong resolve to stop smoking, and viewed this as further motivation to stop smoking. They did not think that it was possible to stop smoking only with the use of medications. This group also reported the side effects and negative experiences in detail

Of the few current smokers who were interviewed, all reported unintentionally taking nicotine substances. They had forgotten that they had applied their nicotine patch, so that the patch’s effect was not felt or was not detected by the patients.

In summary, we can ascertain that the success achieved through pharmaceutical support varied widely among the patients, and they often complained about the side effects and the high cost of this form of treatment. Nevertheless, pharmaceutical support was considered helpful in reducing smoking cravings.

### Experiences with behavioural support

Some patients had also tried reading self-help books, attending smoking cessation seminars, and participating in self-help groups. Patients did not find self-help books helpful; because of their lack of appeal, the patients considered them unsuitable.

*“I understood what was written there, but I didn’t have the feeling that it also applied to me. So now, to me, yes? Well, everybody else can do that, but for me, it won’t work anyway.” (No. 2, m, 62 years)*

Compared to the number of patients who had tried self-help books, more patients had attended smoking cessation seminars, especially during rehabilitation (see Table 3). The smoking cessation seminars during rehabilitation provided practical advice for daily life and information on the smoking cessation process, and they involved the use of visual presentation. The group cohesion that developed among the participants of these seminars was considered helpful. Moreover, this finding indicated that the smokers wished support from other smokers who were in the same situation, and that strong group cohesion was motivational for the smokers.

**Table 3:** Information about behavioural therapies

No.	Have heard about…		Have used…	
	Individual therapy	Group therapy	Individual therapy	Group therapy
1	✓	✓	X	✓
2	✓	✓	X	✓
3	✓	✓	X	X
4	✓	✓	✓	✓
5	✓	✓	X	X
6	✓	✓	X	X
7	✓	✓	✓	X
8	X	✓	X	✓
9	X	✓	X	✓
10	✓	✓	X	X
11	X	X	X	X
12	X	X	X	X
13	X	X	X	n.a.
14	X	✓	X	✓
15	X	X	X	X
16	X	✓	X	X
17	✓	✓	✓	X
18	X	✓	n.a.	X

✓ have heard about the therapies or have used it

X not known before or not used before

*n.a.* not available

*“Yeah … I enjoyed the group and that the group stuck together – the whole group really stuck together – we also stuck to doing what the therapist said.” (No. 14, f, 56 years)*

Nevertheless, some patients felt fearful and ashamed when they reported that their attempted smoking cessation was unsuccessful. These patients also mentioned this when discussing their experiences with self-help groups (only some patients had participated in self-help groups). This topic was not mentioned by the smokers who reported to be part of a strong group cohesion. One assumption is that the strong group cohesion reduced fear and/or shame, and therefore, increased the effectiveness of the program.

*“Besides being terribly ashamed of yourself in front of the others for having smoked again, it brought nothing.” (No. 4, m, 50 years)*

In this context, the patients felt that a mutual exchange of experiences was extremely important in motivating a person to stop smoking, and that peer pressure increased this motivation. Therefore, they preferred that the atmosphere during self-help groups and smoking cessation seminars be very friendly and one that equips the participants with a sense of confidence.

Patients also mentioned that the other limitations of smoking cessation seminars and self-help groups were the lack of flexibility in the organisational structure, and that their content was often illogical. In particular, patients felt that the moderators were not well qualified. Therefore, they did not consider these meetings effective, as they failed to help them to achieve tobacco abstinence.

*“… Are you going to do the ‘non-smoker’ course? Oh no, not again.” No concept. One simply has to tick off. You hear the same thing three times. The way they do it in the rehabilitation clinics is senseless and brainless.” (No. 4, m, 50 years)*

The comparison between the patients who were current smokers and those who had stopped smoking showed that the ex-smokers were better informed about the provision of smoking cessation seminars and knew about possible subsidies by health insurance. The current smokers did not seem to be self-motivated to become informed about smoking cessation seminars.

*“The reporting requirement is missing. As the affected party, one does not get any information on the options one has. This is serious, which is what always makes me a little nervous. Which makes you have a bad attitude and then you resign and at some point you say: ‘Then I will not do it anymore!’ That is eventually the result, which sounds sad. But that’s how it is.” (No.3, m, 59 years)*

*“Um i am, I researched on the internet. I have spent enough time in the rehabilitation centres and then I met the ‘Happy non-smokers’.” (No. 8, f, 56 years)*

Based on the experiences that patients had with smoking cessation seminars during rehabilitation or self-help groups, most of them wished to participate in an entire rehabilitation program only for smoking cessation. They preferred a program based on the existing treatment modalities for alcoholics and drug addicts located in therapy centres, rather than in homes, for a minimum of 1 week and a maximum of 3 months.

*“I found it really terrible that for alcoholics but also for, what do I know – drug addicts etc. There is only one detox method – one option for detox – where they can say ‘I’m going to some clinic and I’m going to stay there for 6 weeks or 3 months, or what do I know?” (No. 1, f, 53 years)*

A rehabilitation program must have certain characteristics that are important, so that patients will participate in it. These characteristics may be derived from patients’ negative experiences with smoking cessation seminars and their preferences for an ideal smoking cessation program. Therefore, rehabilitation should include comprehensive support, a pleasant atmosphere, a combination of medication support, a comprehensive sport and nutrition program, and a wellness area. The patients in the study considered these activities important as they allowed them to take a break from their daily routine and decreased side effects, such as weight gain. The patients’ main expectation for this treatment was that the smoker would learn strategies to stop smoking and manage the withdrawal symptoms in their daily lives.

*“Sport, leisure-time activities … physical activities as in everyday life, in a group, in a center where other activities are possible, with various options … just whatever is useful …” (No. 13, m, 59 years)*

*“Then, I would also very much like a change of scenery … one gets up, one goes to the coffee machine early in the morning – that is compulsory – and then the cup and … and then: Is something missing? The cigarettes, yes. Then I would be able to … block out the environment and all the habits.” (No 5, f, 59 years)*

The patients also felt that comprehensive information about the risks and consequences of smoking should be presented in a visual and drastic form, as part of the consulting services at rehabilitation clinics. According to them, one option was to show videos of COPD patients in the final stages of the disease. Other patients preferred to touch the lung of a deceased smoker.

*“… Then we also spoke about nutrition – that one basically only gains 2.5 to 3.5 k through metabolism alone, and the rest comes from frustrated binging or perhaps from another habit … That was actually a very good thing.” (No. 17, m, 59 years)*

*“Well, it would have to be something very effective. For example, if you go on the Internet and enter “COPD” you will see several people. I downloaded a film from YouTube about a woman who smoked for 35 years. And now her life is hanging on a silken thread … and this woman is not old. Look! That is the final stage!” (No. 1, f, 53 years)*

During the interviews, it became evident that the patients considered it important to have a person who could provide them with emotional support, and who was available at all times of the day, in person, by telephone, or by e-mail. They felt that this person should be part of the rehabilitation process. The patients believed that emotional support was important in reducing their negative attitude, which is one of the side effects of withdrawal.

*“However, one would need to be given the feeling … that if you – what do I know – make several calls a day … it’s also okay and not associated with an unpleasant feeling … according to the motto “Oh! It’s him again!” (No. 8, f, 56 years)*

In summary, behavioral therapeutic measures are useful in supporting the smoking cessation process. Nevertheless, it is important that the major criteria, such as strong group cohesion, a pleasant atmosphere, a moderator who has dealt with the disease, and a memorable and content-related design, be fulfilled. Those requirements may be accomplished through the implementation of a rehabilitation program for smoking addiction with the above named characteristics.

### Experiences with alternative approaches for smoking cessation

In our sample, the patients had tried e-cigarettes, hypnosis, acupuncture, and healing touch therapy (see Table 4). In sum, e-cigarettes is unknown compared to other methods, such as acupuncture and hypnosis. Two patients had used e-cigarettes with nicotine, and both stated that they were uncertain about the risks and long-term side effects of using e-cigarettes. A drawback of e-cigarettes that was identified by the patients was the lack of satisfaction stemming from the fact that one can never ‘finish’ smoking an e-cigarette.

**Table 4:** Information about alternative methods

	Have heard about …				Have used…			
Number	Hypnosis	Acupuncture	E-cigarette	Self-help books and CDs	Hypnosis	Acupuncture	E-cigarette	Self-help books and CDs
1	✓	✓	X	✓	X	✓	X	X
2	X	✓	X	✓	X	✓	X	✓ (book and CD)
3	✓	✓	✓	✓	X	X	X	✓ (book)
4	X	✓	X	✓	✓	✓	X	✓ (book and diary)
5	✓	✓	✓	✓	X	X	X	X
6	✓	✓	X	✓	X	X	n.a.	X
7	X	X	X	X	X	X	X	X
8	✓	✓	X	✓	✓	✓	n.a.	✓ (CD)
9	X	X	X	X	X	X	X	X
10	X	X	X	X	X	X	X	X
11	X	X	X	X	X	X	X	X
12	✓	✓	X	✓	X	✓	n.a.	✓ (book)
13	✓	✓	X	X	X	X	n.a.	X
14	X	X	✓	X	X	X	n.a.	X
15	X	X	✓	X	X	X	✓	n.k.
16	X	✓	X	X	X	X	X	X
17	✓	✓	X	X	X	X	X	X
18	✓	X	✓	X	X	X	✓	X

✓ have heard about the therapy or have used it

X not known before or not used before

*n.a.* not available

*“We have also tried it, but e-cigarette could be more dangerous than normal cigarettes, because it has not been researched.” (No. 18, f, 64 years)*

The advantages of e-cigarettes that were mentioned were their reasonable cost and their ability to serve as a substitute for cigarettes because they provided the patients with the sensation of smoking actual cigarettes. The patients also stated that the e-cigarettes were better for usage around family and friends because they reduced exposure to passive smoke and smelled less than normal cigarettes.

*“That way I still have the feeling that I am smoking, but without having bad breath, bothering others, and poisoning anyone.” (No. 15, m, 59 years)*

Only two of the patients had undergone hypnosis for smoking cessation; however, more patients had heard about experiences with hypnosis from family and friends. Neither of the two patients were satisfied with hypnosis, as it was unsuccessful in helping them to stop smoking. One patient managed to abstain from smoking for 4 weeks after hypnosis but long-term abstinence was not realized. Nevertheless, hypnosis did help the patients learn strategies, such as distraction and self-motivation techniques (e.g. autosuggestion) to combat tobacco cravings.

*“Yes, we could then also visualise … so, first of all, the smoking behaviour, why we smoke. But he packaged it more like, yes – like an art show. You see? It was … it went easily, but somewhere along the line it stopped anyway. Also lots of positive assimilation. These also have an influence… ” (No. 8, f, 56 years)*

The main reasons the patients did not opt for hypnosis was its high cost and their unwillingness to spend so much money on a therapy with a low probability of success. Most of the patients were not convinced that hypnosis could help a person stop smoking.

*“And I said that if I went there five or six times, and then had to pay, what do I know, 1000 EUR. And it didn’t work. Then I would say, I don’t know, one would have to give me a guarantee and say, “So, now you have really done it!” That would be okay for me. Then I would do it.” (No. 6, f, 58 years)*

The patients were disappointed because they experienced no benefits from the therapy. Three patients had undergone acupuncture therapy and most of the others had heard about it from family and friends. The patients reported that acupuncture helped to reduce smoking cravings only for a few days, and one patient complained of experiencing a burning sensation in the ears.

*“Well, I just think it did not bring very much – there was 1 or 2 days.” (No.12, f, 59 years)*

Given the fact that COPD patients are highly dependent on cigarettes, some of them visited healers who used an energy-based approach to healing, called healing touch. This type of therapy involves practitioners’ use of their hands to influence the human energy system. Most of the patients believed that the healers were interested only if the person who visited them believed in supernatural powers. The patients stated that the healer helped them in terms of increasing their motivation to stop smoking, although only for a limited period. One patient mentioned that the motivation to stop smoking lasted for only 1 day.

*“Oh, well. That’s the way it was. So. Perhaps it was all nonsense. But this nonsense must have been so credible that many people perhaps tried just as hard as I did.” (No. 3, f, 54 years)*

Patients reported different experiences with the different therapies, and achieved different results. Some patients were unable to stop smoking, whereas others managed to stop smoking for 4 weeks or longer.

Only one current smoker had experience with an alternative approach (e-cigarette) for smoking cessation, whereas no other current smokers wanted to use alternative approaches because of the bad experiences of friends and families. They all believed what other people said and thought that the methods were too expensive.

**Fig. 1:**
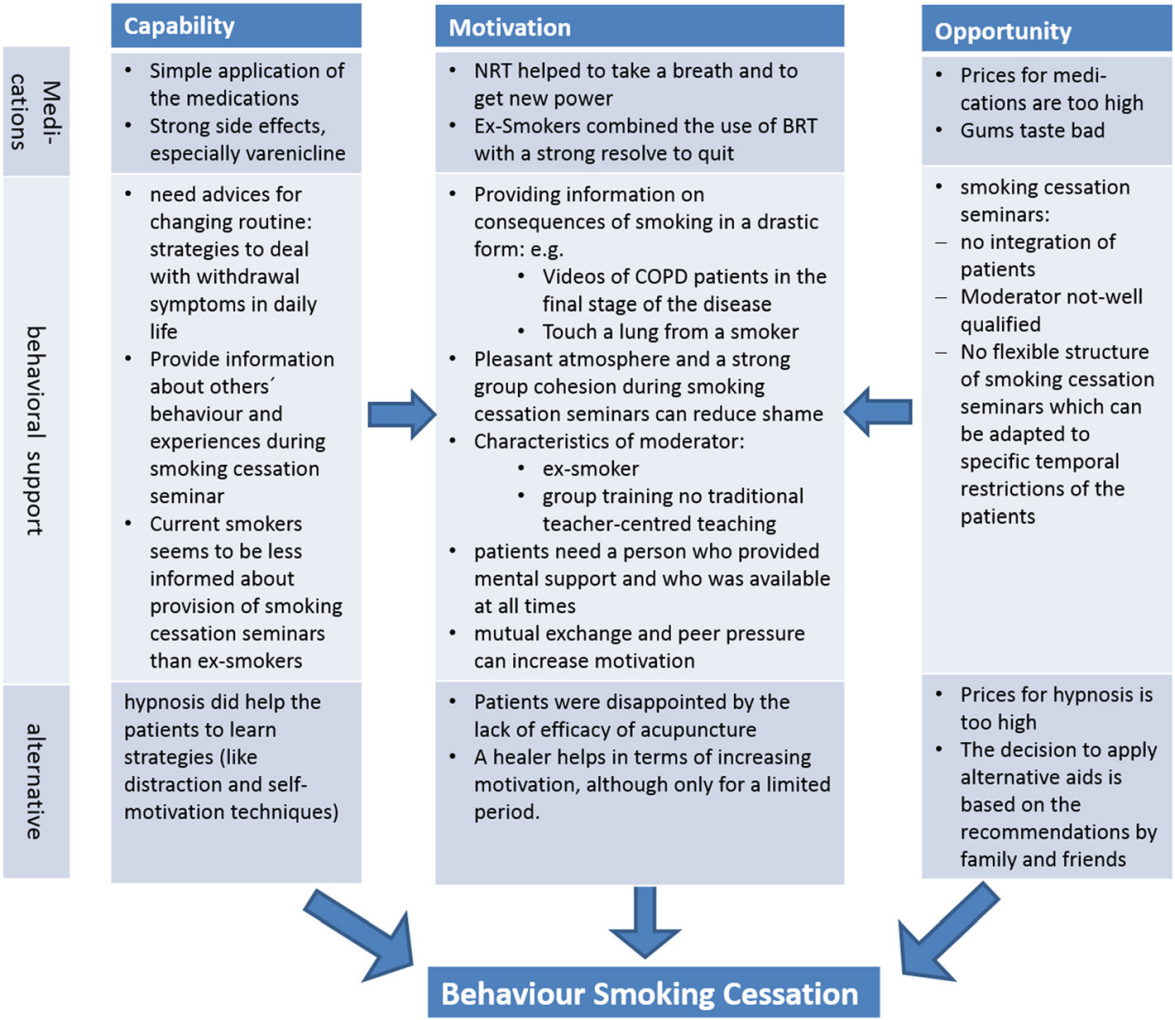
Results according to the three factors of the COM-B Model

All different experiences were sorted using the factors of the COM-B framework. Figure 1 shows the main results of the analysis of the three different smoking cessation methods on each of the factors of capability, opportunity, and motivation.

## Discussion

The COPD patients had varying experiences with different smoking cessation therapies. By analysing the interviews, we identified the patients’ positive and negative experiences with the major smoking cessation therapies currently available in Germany, and their preferences for the improvement of the smoking cessation process. In the following section we compared the results according to the COM-B Model with the existing literature.

### Medications

Patients reacted favorably to taking medications for support during the smoking cessation process, especially to take a deep breath and thereby attenuate smoking cravings. However, they often criticized the medications’ high costs and side effects. In Germany, medications for smoking cessation are excluded by law (§34 SGB V) from reimbursement by compulsory health insurance [13]; therefore, patients have to pay for these medications themselves. In Europe, most of the countries reimburse at least one form of counselling but only some countries, (e.g. the UK) reimburse medications for smoking cessation [14]. The costs for both therapies, NRT and cessation programs are covered in the United Kingdom, Ireland, Denmark and France [15, 16]. A study from the Netherlands assessed the impact of changes in reimbursement policy on the use of and adherence to these medications [17]. The results showed that reimbursement of medications was associated with increased adherence to medication, which should increase the likelihood of the medication’s effectiveness. A cost effectiveness analysis by Cadier et al. show that free access to cessation treatment in the France health care system is cost effective and may result in cost savings compared to a coverage of 50€ [18]. These results indicated that providing financial support to COPD patients was an option for improving their motivation during the smoking cessation process, could change their smoking behavior and could reduce health care costs in the future.

The reported side effects of the therapies had a negative influence on the patients’ likelihood of using these medications, and therefore, a negative influence on the patients’ motivation. Most side effects were reported with Vareniclin. Nausea and the more rarely reported side effects of deafness and loss of body control were significant disadvantages in this group. The FDA also warns against side effects that influence behavior hostility, agitation and depressed mood [19]. For the different forms of NRT less side effects were reported. By using nicotine patches the tingling sensation in the hands and the daily changes in the skin that came into contact with the patch were reported negatively. None of the patients in this study stopped using nicotine patches due to the side effects. Other results were reported by Eklund et al. [9]. Some of the patients even stopped using the nicotine patch due to its powerful side effects. The patients in our study also complain about the bad taste of chewing gum. This could be confirmed by other studies that also provided evidence regarding the unpleasant taste of nicotine chewing gum [20–22]. Nevertheless the patients interviewed in this study thought that NRT have few side effects and could be useful to stop craving. The safety of the different forms of NRT and small side effects were also confirmed by other studies and it is therefore the first line [23, 24].

Our results showed that the use of NRT alone was not an effective way to quit smoking. It was necessary to combine the use of NRT with a strong resolution to quit. The explanation is that NRT motivated the patients because it helped them take a deep breath. Nevertheless, a study by van Eerd et al. reported that cravings did not stop with the aid of NRT [8], and stopping cravings is a prerequisite to using medications for motivational support.

### Behavioral support

In addition to the medications, patients had experiences with behavioral support in the form of smoking cessation seminars. The seminars were the most frequently discussed method during the interviews. In particular, the structure and content of the smoking cessation seminars that were identified during the interviews influenced the patients’ motivation to stop smoking. Most of the smoking cessation seminars in Germany are part of different rehabilitation programs, which are reimbursed by self-insurance. One component of this rehabilitation program is group therapy for smoking cessation. As the patients in this study frequently complained about the lack of qualified moderators in the group therapy sessions, it was not comparable with the holistic approaches to smoking cessation required in rehabilitation programs for smokers. One possible reason for the patients’ lack of acceptance of group therapy might have been its uncomfortable setting. According to the patients, the smoking cessation seminars should fulfil certain criteria, for example, education about the health risks of smoking, providing information about reducing relapse risk [25], and certification of the trainers. These conditions should provide the patients with the possibility and capability of increasing their motivation (see Fig. 1). Nevertheless, the guidelines for smoking cessation seminars in Germany have no specific information on the manner in which the content should be presented. Additionally, the recommendations of the UK’s National Institute for Health and Care Excellence for group behavior therapies are rather general [26]. They consist of scheduled meetings where people who smoke receive information, advice, and encouragement, and some form of behavioral intervention. Therefore, the trainers are free to choose their own teaching style (e.g. traditional teacher-centered instruction and training in group settings). Additionally, there is inadequate information about strategies for relapse prevention.

One way to improve the quality of these seminars is to revise the guidelines and add requirements that the seminars should fulfil. These requirements should include the preferences expressed by the patients, such as visual presentations of the consequences of smoking in the form of videos. Another qualitative study by Bethea et al. revealed that the use of visual media was an important method for relaying information [27]. Therefore, monitoring the quality of these seminars should improve their quality and acceptance by smokers, as an effective way to help them stop smoking. Improvements in these seminars should result in smokers’ increased motivation during smoking cessation. Also other authors mentioned that patients with COPD need a higher level of support for smoking cessation [23, 28]. This is clearly apparent in the example of hospitalization compared with usual care. After 1 year the authors calculated a quit rate of 52 % for hospitalization compared to 7 % for usual care. These results underpin the discussion about the implementation of rehabilitation programs for smoking cessation.

### Alternative methods

The alternative methods for quitting smoking were generally described as ineffective. This finding is consistent with a Cochrane review of hypnosis therapy [29]. Nevertheless, our study’s results showed that hypnosis helped patients learn new strategies for self-motivation techniques, and therefore, improved their capabilities. Because only a small number of interviewed patients had experience with alternative methods, no further results were reported.

### Strengths and limitations

Overall, this study has several strengths and limitations. It includes only COPD patients from Germany; thus, the obtained information on smoking cessation programs is only from Germany. Nevertheless, the patients’ experiences with medications might be transferred to other European countries. In addition, the guidelines for the content of smoking cessation seminars seemed to be comparable between some countries (e.g. UK and Germany). The interviews were conducted via telephone, and, in some cases, personally. The patients interviewed via telephone may have failed to provide complete answers or may not have answered questions in the same manner as they would have in the case of a personal interview. However, these patients provided a detailed report on the smoking cessation process. We also integrated more ex-smokers than current smokers so that the experiences of ex-smokers may be overrepresented. Nevertheless this study provides some insight into the differences between patients with COPD who are current and former smokers, but in this case, further research is necessary. This study also adds to the existing knowledge about the experiences of patients with COPD and provides further insight into the problems of smoking cessation seminars and the improvements needed for their acceptance by the patients. This study provides COPD patients’ impressions of their experiences with alternative smoking cessation therapies, such as hypnosis and acupuncture. In summary, our results provide valuable information about patients’ preferences for smoking cessation methods and can help improve the smoking cessation process.

## Conclusion

We identified several different experiences of patients with COPD with different smoking cessation therapies. The findings of this study provide us with a deeper understanding of which experiences made it easy and which made it difficult for patients to change their smoking behaviors. Based on these problems, some improvements in smoking cessation programs are necessary to ensure that patients have better experiences and increased motivation.

We identified experiences with medications, smoking cessation seminars, and alternative methods of smoking cessation. High prices and powerful side effects made it difficult for patients to use medications for smoking cessation. Nevertheless, the medications helped the patients to take a deep breath. Based on these experiences, providing financial support for buying these medications could reduce the barriers to smoking cessation. The experiences with smoking cessation seminars showed that the content, structure, and seriousness of these seminars negatively influenced the patients’ motivation to stop smoking. Therefore, improvements in the rules for design, implementation, and supervision of smoking cessation seminars, and the integration of rehabilitation clinics, especially for smoking cessation, should be discussed. The results showed that some negative experiences with smoking cessation were explained by the patients’ lack of motivation or determination. Other experiences, such as the structure of smoking cessation seminars or the high price for pharmaceuticals should be addressed through policy changes, which should result in patients’ higher motivation to quit smoking.

This study adds further information about the experiences that influence the quitting process of patients with COPD and their preferences for improvement. Further studies of the differences between patients with COPD who are current and former smokers are needed. The findings could provide further insight into the factors that determine successful cessation.

## Abbreviations

COPD: Chronic obstructive pulmonary disease
NRT: Nicotine replacement therapy
